# Incidence of acquired demyelinating syndromes of the CNS in Dutch children: a nationwide study

**DOI:** 10.1007/s00415-012-6441-6

**Published:** 2012-02-17

**Authors:** I. A. Ketelslegers, C. E. Catsman-Berrevoets, R. F. Neuteboom, M. Boon, K. G. J. van Dijk, M. J. Eikelenboom, R. H. J. M. Gooskens, E. H. Niks, W. C. G. Overweg-Plandsoen, E. A. J. Peeters, C. M. P. C. D. Peeters-Scholte, B. T. Poll-The, J. F. de Rijk-van Andel, J. P. A. Samijn, I. N. Snoeck, H. Stroink, R. J. Vermeulen, A. Verrips, J. S. H. Vles, M. A. A. P. Willemsen, R. Rodrigues Pereira, R. Q. Hintzen

**Affiliations:** 1 MS Centre ErasMS, Department of Neurology, Room Ee 2230, Erasmus MC, PO Box 2040, 3000 CA Rotterdam, The Netherlands; 2 Department of Neurology, University Medical Center Groningen, Groningen, The Netherlands; 3 Department of Pediatrics, Rijnstate Hospital, Arnhem, The Netherlands; 4 Department of Child Neurology, Neuroscience Campus Amsterdam, VU Medical Center, Amsterdam, The Netherlands; 5 Department of Pediatric Neurology, University Medical Center Utrecht, Utrecht, The Netherlands; 6 Department of Neurology, Leiden University Medical Center, Leiden, The Netherlands; 7 Department of Pediatric Neurology, Emma Children’s Hospital/AMC, Amsterdam, The Netherlands; 8 Department of Neuropediatrics, Juliana Children’s Hospital/Haga Hospital, The Hague, The Netherlands; 9 Department of Neurology, Amphia Hospital, Breda, The Netherlands; 10 Department of Neurology, Maasstad Hospital, Rotterdam, The Netherlands; 11 Department of Neurology, Canisius-Wilhelmina Hospital, Nijmegen, The Netherlands; 12 Department of Neurology, University Hospital Maastricht, Maastricht, The Netherlands; 13 Department of Pediatric Neurology, Radboud University Nijmegen Medical Centre, Nijmegen, The Netherlands; 14 Nederlands Signaleringscentrum Kindergeneeskunde (NSCK), TNO Quality of Life, Leiden, The Netherlands; 15 Department of Paediatric Neurology, Erasmus MC/Sophia Children’s Hospital, Rotterdam, The Netherlands

**Keywords:** Multiple sclerosis, Demyelinating diseases, Incidence, Pediatric

## Abstract

Acquired demyelinating syndromes (ADS) can be a first presentation of multiple sclerosis (MS) in children. The incidence of these disorders in Europe is currently unknown. Children (<18 years old) living in the Netherlands who presented with ADS were included from January 1, 2007 to December 31, 2010 by the Dutch pediatric MS study group and the Dutch surveillance of rare pediatric disorders. Demographic and clinical data were collected. Eighty-six patients were identified over 4 years, resulting in an incidence of 0.66/1,00,000 per year. Most patients presented with polyfocal ADS without encephalopathy (30%), followed by polyfocal ADS with encephalopathy (24%), optic neuritis (ON, 22%), monofocal ADS (16%), transverse myelitis (3%), and neuromyelitis optica (3%). Patients with polyfocal ADS with encephalopathy were younger (median 3.9 years) than patients with ON (median 14.6 years, *p* < 0.001) or monofocal ADS (median 16.0 years, *p* < 0.001). Patients with polyfocal ADS without encephalopathy (median 9.2 years) were also younger than monofocal ADS patients (median 16.0 years, *p* < 0.001). There was a slight female preponderance in all groups except the ON group, and a relatively large number of ADS patients (29%) reported a non-European ancestry. Familial autoimmune diseases were reported in 23%, more often in patients with relapsing disease than monophasic disease (46 vs. 15%, *p* = 0.002) and occurring most often in the maternal family (84%, *p* < 0.001). During the study period, 23% of patients were subsequently diagnosed with MS. The annual incidence of ADS in the Netherlands is 0.66/1,00,000 children/year. A polyfocal disease onset of ADS was most common.

## Introduction

In the last decade, knowledge about pediatric multiple sclerosis (MS) and other demyelinating diseases of the CNS has increased considerably. As a group, these first immune-mediated demyelinating events of the CNS are referred to as acquired demyelinating syndromes (ADS) [1]. They share common clinical characteristics and they can all represent a first episode of MS. Due to increased awareness among clinical professionals, these diagnoses are likely to be made more often. At present, only one prospective study reported about the incidence of ADS [1]. Other available incidence studies had a retrospective design [2] or focused on subgroups of ADS, like acute disseminated encephalomyelitis (ADEM) and MS [3].

In 2007, we started a nationwide prospective surveillance study to define the incidence of ADS in the Netherlands. We used the network of collaborators of the Dutch pediatric MS study group and participated in a nationwide surveillance program to detect rare pediatric diseases in which all Dutch pediatricians are involved. We here describe the incidence as well as clinical and demographic characteristics of children with ADS in the Netherlands.

## Methods

### Patient inclusion

Children younger than 18 years and living in the Netherlands and suspected of a first inflammatory demyelinating event of the CNS who were detected by our surveillance from 2007 to 2010 were included in this study.

Diagnoses were made in accordance to the criteria proposed by the International Pediatric MS Study Group (IPMSSG) [4]. Based on clinical and MRI data, patients were divided into six ADS groups: (1) optic neuritis (ON), (2) transverse myelitis (TM), (3) monofocal ADS (mono ADS), (4) polyfocal ADS (poly ADS) without encephalopathy, (5) polyfocal ADS with encephalopathy, and (6) neuromyelitis optica (NMO). We avoided the term ADEM [5], because of inconsistent use of this term in previous studies and chose to define this group more transparently as ‘poly ADS with encephalopathy’ according to the definition proposed by the IPMSSG.

A diagnosis of MS was made in case of a second demyelinating attack of the CNS with clinical and/or MRI evidence of a new lesion localization at least 1 month after onset. A patient who presented with poly ADS with encephalopathy, required at least two new episodes without encephalopathy, at least 3 months after onset, for a diagnosis of MS [4].

Patients were excluded if another cause of the neurological symptoms was demonstrated, including infectious, metabolic, toxic, or systemic immunological causes.

The patients were identified using two methods, in order to reach nationwide inclusion of patients:

- In the PROUDkids study (PRedicting the OUtcome of a Demyelinating event in children) pediatric neurologists of the eight Dutch academic hospitals and of five non-academic neuropediatric hospitals are involved. The aim of this study is to investigate prognostic factors that predict MS diagnosis in children after ADS.
- The NSCK (Netherlands Paediatric Surveillance Unit) reaches all Dutch pediatricians monthly by e-mail and aims to provide insight in the epidemiology of rare pediatric diseases in the Netherlands. Pediatricians were asked to report whether they did or did not see a patient suspected of a CNS inflammatory demyelinating disease.

Patients were included in the study after written informed consent was obtained from parents and patients older than 12 years. A standardized scoring template was used to gather demographic and clinical information of all reported patients. Demographic data consisted of sex, date, and place of birth, ethnic background and family history in first- and second-degree relatives. Country of birth and ancestry was asked to both the child and his parents: patients with at least one parent of non-European origin were classified as of non-European origin. Whenever possible, we asked both parents about their family history with emphasis on familial autoimmune diseases like autoimmune thyroid disease, rheumatoid diseases, and type I diabetes. Clinical data consisted of date of disease onset, clinical symptoms, concomitant diseases, infection or vaccination in preceding 4 weeks, hospitalization, and treatment. MRI, blood, and CSF results were also collected for diagnostic evaluation. Follow-up data were provided by the treating physician and by telephone interview of the parents at least once 2 years after disease onset. Clinical records were then evaluated. We assessed whether diagnoses changed during follow-up and the patient had to be excluded. We also assessed whether a final diagnosis of MS could be made.

The clinical, laboratory, and imaging data were reviewed (by IAK) in order to ensure that patients met the inclusion criteria and to diagnose them appropriately. This study was approved by the Medical Ethical Committees of the Erasmus University Medical Centre in Rotterdam and of the other participating centers.

### Analysis

Demographical data of the Dutch population were derived from Statistics Netherlands [6]. Statistical analysis was performed using SPSS 17.0. The Kruskal–Wallis and Chi-square tests were used to test differences in clinical and demographic characteristics between the six groups. Mann–Whitney *U* tests were used to follow-up differences in numerical data between groups, whereas categorical data were compared using Chi-square or Fisher’s exact tests. A Bonferroni correction was applied, so all effects are reported at a 0.0083 significance level.

The Chi-square test was also used to compare the ethnic background of our patients with the Dutch population, to compare the autoimmune family history between monophasic and relapsing patients, and to compare seasonal distribution in the entire group. Results were considered significant if *p* < 0.05. Unknown or not reported data in all groups were removed from the analyses.

## Results

From January 1, 2007 to December 31, 2010, 111 children were reported. One child and her parents refused inclusion. Eighty-six of the reported patients met the inclusion criteria and were analyzed. Twenty-four reported patients (22%) had an alternative diagnosis and were subsequently excluded. The final diagnoses of the excluded patients are listed in Table 1. The correct diagnoses were made by laboratory tests, blood and CSF studies, MRI/MR angiography, as well as clinical course and response to treatment.

**Table 1:** Diagnosis of the excluded patients

Diagnosis	*n* = 24
Infectious disease	11
Viral encephalitis	6
Postinfectious TM	3
Meningitis	2
Systemic inflammatory or autoimmune disease	6
(Cerebral) vasculitis	3
Celiac disease	1
Susac’s syndrome	1
Hashimoto encephalopathy	1
Mitochondrial disease	4
Leber hereditary optic neuropathy (LHON)	1
Other	3
Neoplastic disease	2
Posterior reversible encephalopathy syndrome (PRES)	1

Given the number of children in the Netherlands younger than 18 years in 2007 (3,360,433), 2008 (3,344,945), 2009 (3,329,173), and 2010 (3,314,663) [6], the average incidence of ADS is 0.66/1,00,000 Dutch children per year (0.60/1,00,000 in 2007, 0.60/1,00,000 in 2008, 0.72/1,00,000 in 2009, 0.72/1,00,000 in 2010).

Thirty percent of patients presented with poly ADS without encephalopathy, 24% with poly ADS with encephalopathy, 22% with ON, 16% with mono ADS, 3% with TM, and 3% with NMO.

**Fig. 1:**
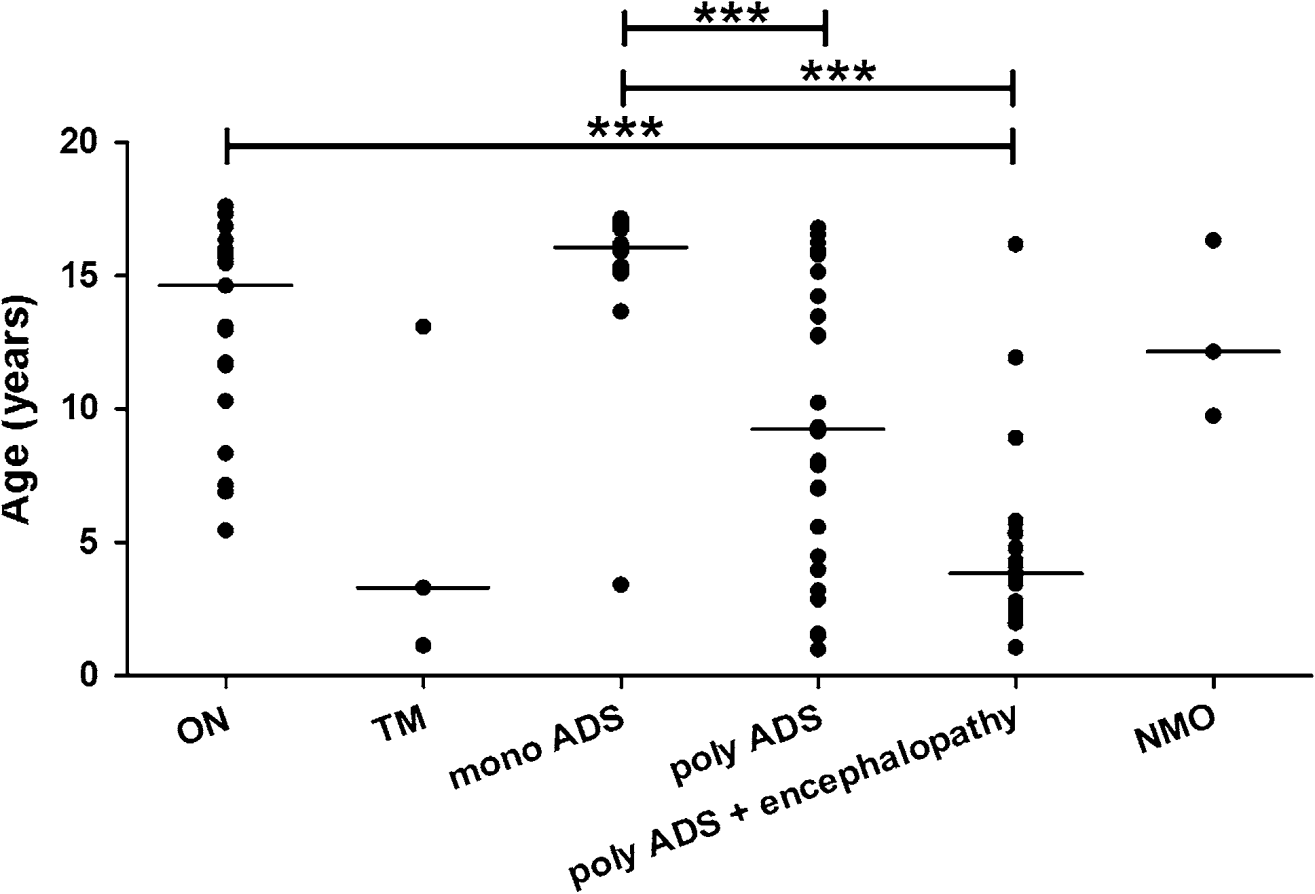
Age distribution of the patients, categorized by clinical presentation. The median age per group is shown. The *horizontal lines* above the groups indicate statistical differences between the groups (Mann–Whitney *U* test, *** *p* < 0.001). *ON* optic neuritis, *TM* transverse myelitis, *ADS* acquired demyelinating syndrome, *mono* monofocal, *poly* polyfocal *NMO* neuromyelitis optica

Figure 1 shows the distribution of the patients’ age at clinical presentation. Children with poly ADS with encephalopathy were younger (median 3.9 years) than children with ON (median 14.6 years) (*U* = 29, *p* < 0.001) and children with monofocal ADS (median 16 years) (*U* = 22, *p* < 0.001). Also children with poly ADS without encephalopathy (median 9.2 years) were younger than children with mono ADS (*U* = 63, *p* < 0.001). No significant differences were observed between the other groups.

The demographic characteristics of the included patients are summarized in Table 2. The female–male distribution was similar between the groups. After stratification of all children with ADS in a group younger (*n* = 41) and a group older than 10 years (*n* = 45), we also found a similar female:male ratio of 1.3:1 in the younger and of 1.1:1 in the older group.

**Table 2:** Demographic characteristics of all patients, categorized by clinical presentation

	ON (*n* = 19)	TM (*n* = 3)	Mono ADS (*n* = 14)	Poly ADS without encephalopathy (*n* = 26)	Poly ADS with encephalopathy (*n* = 21)	NMO (*n* = 3)	*p* value^a^	All (*n* = 86)
Female:male	0.9:1	2:1	1.3:1	1.4:1	1.1:1	2:1	0.97	1.2:1
European ethnicity, *n* (%)	12 (63)	2 (67)	6 (43)	16 (62)	15 (71)	3 (100)	0.70	54 (63)
Non-European ethnicity, *n* (%)	5 (26)	1 (33)	5 (36)	9 (35)	5 (24)	0	0.70	25 (29)
Middle-Eastern	2 (11)	0	0	2 (8)	1 (5)	0		5 (6)
African	1 (5)	0	4 (29)	2 (8)	1 (5)	0		8 (10)
Middle-American	1 (5)	0	1 (7)	1 (4)	0	0		3 (4)
Asian	0	0	0	1 (4)	1 (5)	0		2 (2)
Mixed^b^	1 (5)	1 (33)	0	3 (12)	2 (10)	0		7 (8)
Unknown ethnicity	2 (11)	0	3 (21)	1 (4)	1 (5)	0	0.42	7 (8)
Place of birth: outside Europe, *n* (%)	1 (5)	0	1 (7)	2 (8)	0	0	0.81	4 (5)
Autoimmune family history, *n* (%)	1 (5)	0	7 (50)	8 (31)	3 (14)	1 (33)	0.05	20 (23)
MS	1 (5)	0	1 (7)	0	0	1 (33)	0.18	3 (3)
Other autoimmune disease	0	0	6 (43)	8 (31)	3 (14)	0	0.02^c^	17 (20)
Unknown family history	5 (26)	2 (67)	3 (21)	7 (27)	7 (33)	0	0.54	24 (28)

*ON* optic neuritis, *TM* transverse myelitis, *ADS* acquired demyelinating syndromes, *mono* monofocal, *poly* polyfocal, *NMO* neuromyelitis optica, *MS* multiple sclerosis

^a^Patients between the six ADS groups are compared (*χ* ^2^ test for categorical data)

^b^Mixed ethnicity: one parent of European origin and one parent of non-European origin

^c^Not significant after Bonferroni correction

Twenty-five patients (29%) were of non-European origin. This proportion was higher than the proportion of children of non-European origin (<18 years old) in the general pediatric population in the Netherlands (16%) [6] (*χ* ^2^ = 13.992, *p* < 0.001). The incidence of ADS in children of European origin is 0.52/1,00,000 per year, in contrast to 1.16/1,00,000 per year in children of non-European origin in the Netherlands. Most of these non-European patients (84%) were born in the Netherlands themselves.

A familial history of autoimmune diseases was present in 23% of all patients. No difference was observed in the presence of autoimmune diseases (including MS) in the first- and second-degree relatives between the six ADS subgroups. Autoimmune thyroid diseases and rheumatoid arthritis were most frequently reported (both in five cases). Only three ADS patients reported MS, all in the maternal family. Familial autoimmune diseases occurred more often in patients with relapsing disease than in patients with monophasic disease (46 vs. 15%, *χ* ^2^ = 9.51, *p* = 0.002). A maternal family history of autoimmune diseases was much more frequent than a paternal family history of autoimmune diseases (84 vs. 16%, *p* < 0.001).

In the ADS patients, we found no difference between a disease onset in winter (36%), spring (21%), summer (24%), or autumn (19%).

Table 3 shows the other clinical characteristics at first demyelinating attack of the included patients. The differences in preceding infection between the groups did not reach significance. Only one child received a vaccination (measles mumps and rubella vaccine) before onset of a polyfocal disease with encephalopathy.

**Table 3:** Clinical characteristics of all patients, categorized by clinical presentation

	ON (*n* = 19)	TM (*n* = 3)	Mono ADS (*n* = 14)	Poly ADS without encephalopathy (*n* = 26)	Poly ADS with encephalopathy (*n* = 21)	NMO (*n* = 3)	*p* value^a^	All (*n* = 86)
Previous infection, *n* (%)	1 (5)	2 (67)	1 (7)	9 (35)	10 (48)	0	0.01^b^	23 (27)
Previous vaccination, *n* (%)	0	0	0	0	1 (5)	0	0.70	1 (1)
Cerebral MRI pathology, *n* (%)	8 (42)	0	14 (100)	23 (88)	21 (100)	1 (33)	**<0.001** ^**c**^	67 (78)
Follow-up time, months, median (range)	14.3 (1.1–42.3)	9.9 (2.3–15.6)	21.4 (2.8–45.9)	12.5 (1.5–51.6)	13.2 (0.3–44.8)	7.3 (1.8–19.1)	0.66	12.9 (0.3–51.6)
Relapsing disease, *n* (%)	6 (32)	0	7 (50)	9 (35)	2 (10)	0	0.07	24 (28)
MS, *n* (%)	4 (21)	0	7 (50)	9 (35)	0	0	0.01^b^	20 (23)
Second clinical attack ≤2 years, *n* (% of relapsing patients)	5 (83)	0	6 (86)	5 (56)	2 (100)	0	0.37	18 (75)

*ON* optic neuritis, *TM* transverse myelitis, *ADS* acquired demyelinating syndromes, *mono* monofocal, *poly* polyfocal, *NMO* neuromyelitis optica, *MRI* magnetic resonance imaging, *MS* multiple sclerosis

^a^Patients between the six ADS groups are compared (*χ* ^2^ test for categorical data and Kruskal–Wallis test for numerical data)

^b^Not significant after Bonferroni correction

^c^Mono ADS compared to ON (Fisher’s exact test, *p* = 0.001), poly ADS with encephalopathy compared to ON (Fisher’s exact test, *p* < 0.001)

All patients (except one child with TM) underwent brain MR imaging. Sixty-seven patients with ADS presented with demyelinating lesions on brain MRI. Cerebral MRI lesions were more frequent in children with mono ADS (Fisher’s exact test, *p* = 0.001) and poly ADS with encephalopathy (Fisher’s exact test, *p* < 0.001) as compared to children with ON.

Twenty-eight percent of the patients experienced at least one relapse. One patient experienced two episodes of ON after a poly ADS with encephalopathy (with resolution of previous clinical symptoms and MRI abnormalities and without new MRI lesions), one patient suffered from another episode of poly ADS with encephalopathy and two patients had recurrent ON without cerebral MRI lesions. By now, 20 patients have been diagnosed as MS. More patients with a monofocal onset (Fisher’s exact test, *p* = 0.001) or a polyfocal onset without encephalopathy (Fisher’s exact test, *p* = 0.003) were subsequently diagnosed with MS compared to patients with a polyfocal onset with encephalopathy. Five patients were diagnosed as MS based on asymptomatic new MRI lesions. The proportion of patients with a relapsing disease who experienced their second clinical attack within 2 years after onset (excluding patients with only MRI evidence of new disease) was comparable between the different diagnostic groups. Only three children younger than 10 years experienced a second clinical attack. The patients who were subsequently diagnosed with MS were all older than 10 years at the time of their first demyelinating attack.

## Discussion

The incidence of ADS in children in the Netherlands is 0.66/1,00,000 per year. It is difficult to compare this number with the incidence in other countries because only two studies defined and analyzed the incidence of ADS [1, 2] whereas another study focused on solely ADEM and MS [3].

A nationwide prospective study in Canada reported an annual incidence of ADS of 0.9/1,00,000 [1]. Both our study and the Canadian study succeeded to reach nationwide coverage by using recently initiated centralized national databases. The lower incidence observed in the Netherlands may be explained in part by geographical difference and differences in demographic characteristics of the pediatric patients, especially regarding ethnicities. In the Canadian group, 37% of children were first-generation Canadians (i.e., both parents born outside Canada) [1] whereas in the Netherlands this count was 18%.

A recent paper showed that the incidence of ADS in the United States is 1.63/1,00,000 per year [2]. This is much higher than our or the Canadian incidence. The main difference between this US and both our and the Canadian study is the methodology: the US investigators retrospectively searched a large health maintenance organization database in one area. The incidence in this multiethnic cohort of Southern Californian children was then used to extrapolate the incidence in the US. The question can be raised of whether this study population is representative of the entire US. Furthermore, the higher incidence may be due to the large ethnic diversity in this cohort [2].

A nationwide German survey focused on children (<16 years old) with ADEM and MS. They reported an annual incidence of MS of 0.3/1,00,000 [3]. So far, we found an annual incidence of MS of 0.15/1,00,000 in Dutch children. This difference may be caused by the still short follow-up time of the patients in our study. The real incidence of pediatric MS in the Netherlands can only be calculated when the youngest child of our cohort reaches the cut-off age for pediatric ADS of 18 years. The higher incidence of MS in Germany can also be due to their inclusion of both suspected as definite MS patients [3].

A remarkable finding in the German study was the much lower incidence of ADEM (0.07/1,00,000) [3]. The authors speculated that this low number of ADEM patients could be a consequence of geographical differences with other cohorts [3]. Although ADEM was not defined in their study, we found an annual incidence of patients with a polyfocal onset with encephalopathy (ADEM according to the IPMSSG [4]) of 0.16/1,00,000. So we could not confirm this low incidence of ADEM in a geographical comparable area.

In our study, poly ADS without encephalopathy was the most common presentation (30%), followed by poly ADS with encephalopathy (24%) and ON (22%).

TM was very rare in our cohort (3%) in contrast to the Canadian cohort (22%) [1]. The Canadian children with TM differed from the Dutch children with TM: they were much older (mean age at onset of 11 years) and MRI abnormalities (either cerebral or spinal) were present in 91%. So there could be a discrepancy in defining TM. We classified children with TM and clinical and/or MRI evidence of a localization outside the spinal cord as either polyfocal ADS or NMO. Children who were reported but turned out to have a proven acute and active infectious etiology were excluded.

Most patients had a European ancestry. However, the incidence of ADS was twice as high in children of non-European origin as in children of European origin in the Netherlands. Most of these children were born in the Netherlands themselves. Previous studies in Canada and the US also showed that pediatric MS patients more frequently had a non-European ancestry or were non-Caucasian in contrast to adult MS patients, indicating that the pediatric MS population reflects the changing immigration patterns in countries with high MS prevalence. The difference may be explained by the possibility that children with ancestors from a country with low MS prevalence may lack protective genetic factors or are more vulnerable to environmental factors when they grow up in a country with high MS prevalence [2, 7, 8].

Familial MS was reported in only 3% of ADS patients, which is in agreement with previous reports of numbers varying between 3 and 8% [1, 3, 9]. This proportion is higher in retrospective studies on pediatric MS and in studies with longer follow-up durations [10, 11]. Next to MS, we found that other autoimmune diseases in first- and/or second-degree relatives were present in 20% of our patients. Although this number is higher than reported in the Canadian study [1], it is likely to be an underestimation, because the family history was not reported in detail in 28% of patients. Data on the presence of autoimmune diseases in the Dutch pediatric population are not available. We observed that familial autoimmune diseases were especially present in patients with relapsing disease. Studies on the presence of autoimmune diseases in first-degree relatives of adult MS patients show contrasting results [12, 13], but the most recent rigorously performed study suggests that autoimmune diseases are not more frequent in families of MS patients [14].

Of particular interest is the observation that autoimmune diseases were especially frequent in the maternal family. It is not likely that this difference in autoimmune diseases between the mother’s and father’s family is caused by the possibility of only the mothers being the parent interviewed. In all, except for one child, we were able to obtain information on both parents. A maternal parent-of-origin effect has been suggested for MS in adult patients [15–17]. To our knowledge, a possible maternal transmission of autoimmune diseases has not been described before in either pediatric ADS or MS patients or in adult MS patients.

It is plausible to expect that the incidence of ADS we describe in this study is an underestimation because despite all efforts, there are multiple reasons why it is impossible to identify every patient in one country. Theoretically, some ADS patients older than 16 years could have been missed because they may have been directly referred to adult neurologists. We do not expect this to be a large number of patients in our study, as our MS center is a national referral site for both pediatric and adult ADEM, MS, and ADS variants. Secondly, patients with mild or self-limiting symptoms may not be referred to a pediatrician or neurologist at all. Thirdly, ophthalmologists did not participate in this study, so we may have missed a number of ON cases. Still, it is of note that the number of patients with ON we observed is in accordance with a previously reported incidence [1]. Finally, some physicians simply may fail to report patients. However, the response rate in the Dutch NSCK surveillance system is quite high. The number of pediatricians that participated was 86.6% in 2007, 85.5% in 2008, and 84% in 2009 [18]. The strength of our study is that we used two complementary methods to enroll patients. By the NSCK, 44% of the patients were identified and the remaining patients were identified by the PROUDkids study group.

Our study illustrates that other disorders may mimic ADS at first presentation (Table 1). It is important to consider these disorders in the differential diagnosis of ADS, because of potential treatment and prognosis.

ADS can be a challenging diagnosis, and knowing the incidence can increase the awareness of these disorders in children. This is relevant as there are indications of increasing incidence, especially among certain ethnic groups in the Western world.

The research on ADS in the Netherlands is still in progress and the aim is to provide long-term follow-up data in the future.
